# The Use and Abuse of Exercise

**Published:** 1907-02-16

**Authors:** 


### The Use and Abuse of Exercise.

The love of exercise is not inherent in the human adult, though children, like the young of all animals, enjoy playing; with them tissue changes are excessive and beyond their needs, so that they have abundance of superfluous energy. It is otherwise with adults. In these metabolism is either exactly proportional to the needs of the body, or it is insufficient; in the one case the weight remains stationary, in the other it falls. Uncivilised races have enough to do, in obtaining food or withstanding climatic influences, to prevent them feeling the necessity for exercise. It is only since men have thronged into towns and since the multiplication of methods of locomotion have made it cheaper to ride than to walk, that the necessity for regular exercise has become obvious. It does not seem possible to lay down any definite rule as to the amount of exercise required by an individual. Each is a law unto himself, and, more than this, it appears that each generation differs from the preceding in the amount and kind of exercise which is required.

The unbroken traditions of the older university towns, like Oxford and Cambridge, where there has been a residential population somewhat given to writing about itself, tell of the habits of bygone years. At Oxford we know from the diaries of Anthony à Wood and of Thomas Hearne that in the eighteenth century the Masters and Doctors of the university rarely did more than pace the High Street in the afternoon, while in the evening they smoked and drank beer in the alehouses. In the early part of the nineteenth century the walks, now called "grinds," had become more extended, and it was usual for men to go in groups of two or three as far as Headington or Summertown or Wolvercote. But there were no boats, or cricket, or football, and not much riding, except such as was necessary to enable a Fellow on a Sunday to reach the neighbouring parish where he was to preach and dine. Yet this *régime* produced men like Manning, Newman, and Keble; and the walks themselves were a power in the formation of the school of thought associated with these names. They led to conversations and friendly discussions in a manner which has been for ever destroyed by the advent of the bicycle and the motor car. In the present generation the time spent in exercise is shorter than it used to be, but the amount of energy thrown into the shorter time is probably greater than it was formerly.

The physiological object of exercise is to increase the metabolism of the body. Moderate exercise spread over a considerable time leads to growth of the tissues employed, while the waste products are eliminated slowly and efficiently. Excessive exercise, carried to fatigue or compressed into too short a space of time, is bad. It makes a sudden call upon the tissues for which they may be unprepared. They may thus become loaded with the products of their own waste, or the eliminating organs may themselves be insufficient for the extra duties required of them. Many an inflammation of the spinal cord leading to paralysis and death has been traced directly to a long bicycle ride; and the influence of fatigue of the spinal cord in exciting tabes in syphilitic subjects is daily obtaining greater prominence. The cardiac effects of excessive exercise, even in those who have undergone a course of training, are well known, and every boy at a public school is now examined as to his fitness, before he is allowed to take any prominent part in the school games.

It is difficult to forecast what the next generation will cultivate in the shape of exercise. Sport in the form of hunting and shooting will always give opportunity enough to the upper classes in England, but already beagles and coursing, which were the pastimes of the yeoman class, have become as extinct as the yeomen themselves. Cricket, football, and rowing provide an outlet for the high spirits of youth, but there is nothing to take the place of walking for the professional classes and for those whose work keeps them in offices and chambers for long hours. Golf, indeed, has saved the present generation from immediate anxiety as to a sufficiency of exercise, but it is already possible to look forward to a time when even golf links will be too far afield to be reached readily.

